# High-risk HPV E6 induces an aneuploidy-prone chromosome congression defect through destabilization of CENP-E

**DOI:** 10.1128/jvi.00625-26

**Published:** 2026-05-27

**Authors:** Nanami Seshimo, Kanako Hori, Reiko Furuta, Toru Hirota, Ryu-Suke Nozawa

**Affiliations:** 1 Division of Experimental Pathology, Cancer Institute of the Japanese Foundation for Cancer Research, Tokyo, Japan; 2 Division of Diagnostic Pathology, The University of Tokyo Hospital, Tokyo, Japan; 3 Division of Clinical Cytology, Department of Medical Laboratory Sciences, School of Allied Health Sciences, Kitasato University, Kanagawa, Japan; 4 Division of Pathology, Cancer Institute of the Japanese Foundation for Cancer Research, Tokyo, Japan; Tufts University School of Medicine, Boston, Massachusetts, USA

**Keywords:** CENP-E, aneuploidy, E6 oncoprotein, human papillomavirus, chromosomal instability, chromosome segregation

## Abstract

Chromosomal instability is a common feature of malignancies that arises from persistent defects in chromosome segregation during mitosis. The resulting genetic heterogeneity and acquired malignant traits often characterize advanced disease. However, little is known about the mitotic processes underlying the acquisition of chromosomal instability and whether or how they might contribute to the early stages of cancer initiation. Here, we focused on a characteristic chromosome congression defect termed ECAC (Ectopic Chromosome Around Centrosome), which is observed in human papillomavirus (HPV)-positive cervical dysplasia, a premalignant lesion of the cervix. The expression of the HPV oncoprotein E6 induced proteasome-dependent degradation of the kinetochore motor CENP-E in addition to p53. We then identified the region of CENP-E required for E6-dependent degradation and conducted rescue experiments using a degradation-resistant mutant. These experiments demonstrated that CENP-E degradation is the main cause of the ECAC phenotype. Remarkably, the E6-mediated CENP-E destabilization and the resulting induction of ECAC were observed across multiple high-risk HPV genotypes, indicating that these defects represent a broadly shared pathological feature of oncogenic HPV infection. We also found that sustained E6 expression led to the accumulation of aneuploid cell populations during long-term culture. These findings define the molecular basis of ECAC and suggest that persistent chromosome congression defects can initiate chromosomal instability in premalignant cervical lesions.

**IMPORTANCE:**

High-risk human papillomaviruses (HPVs) drive epithelial carcinogenesis through the viral oncoproteins E6 and E7, primarily by disrupting p53- and RB-dependent pathways. While HPV16 E6 has been implicated in chromosome congression defects through destabilization of the mitotic motor CENP-E, it has remained unclear whether CENP-E destabilization is sufficient to induce such defects and whether they are shared across multiple high-risk HPV genotypes. Here, we show in cultured cell models that a degradation-resistant CENP-E mutant suppresses E6-mediated chromosome congression defects and that this mitotic phenotype is consistently observed in cells expressing E6 proteins from multiple high-risk HPV genotypes. Persistence of this mitotic abnormality leads to the accumulation of aneuploid cell populations. These findings link the E6-CENP-E axis to a characteristic mitotic defect in cervical premalignant lesions and suggest that this mitotic abnormality may contribute to the acquisition of chromosomal instability and the development of cervical cancers.

## INTRODUCTION

Faithful chromosome segregation is an essential cellular process for maintaining genome homeostasis. Cancer cells are often defective in chromosome segregation, a phenomenon known as chromosomal instability. Chromosomal instability generates cell populations with diverse chromosomal alterations, increasing intercellular genetic heterogeneity and enabling the selection of cancer cells adapted to tumor microenvironments (1, 2). Through this process, chromosomal instability fuels disease progression across a broad spectrum of cancer types (3–6). However, the mitotic processes that underlie the acquisition of chromosomal instability during early stages of cancer development remain poorly understood.

Cervical cancers are causally linked to infection with the human papillomavirus (HPV). Among approximately 200 HPV genotypes (7), those associated with the cancer development are classified as high-risk HPVs (8). During cervical tumorigenesis, following infection with high-risk HPV, epithelial dysplasia—also termed cervical intraepithelial neoplasia—often precedes cancer initiation. Notably, histopathological examinations of dysplastic lesions have consistently identified mitotic cells in which chromosomes fail to align at the metaphase plate and instead remain near the centrosomes (9–11). This distinctive mitotic abnormality is termed ECAC (Ectopic Chromosome Around Centrosome) (12). These observations have led to the hypothesis that ECAC may contribute to the acquisition of chromosomal instability in premalignant lesions.

Recent studies in HPV-positive head and neck cancers, using patient derived samples and oral keratinocyte models, have shown that HPV16 E6 induces chromosome segregation defects and have implicated CENP-E destabilization in this process (13). However, whether this mechanism also accounts for the ECAC, a characteristic chromosome congression defect observed in high-risk cervical dysplasia, and whether such defects may contribute to the acquisition of chromosomal instability in premalignant lesions, remains to be investigated.

In this study, we aim to investigate the relationship between ECAC and chromosomal instability. We demonstrate that E6-mediated degradation of the mitotic motor CENP-E is a key driver of ECAC. Through a systematic analysis of multiple high-risk HPV genotypes, we establish that ECAC is a mitotic defect commonly induced by the expression of high-risk HPV E6. Notably, longitudinal analyses further reveal that ECAC leads to alterations in chromosome number. These findings provide an insight into how mitotic abnormalities characteristic of premalignant lesions may contribute to the acquisition of chromosomal instability.

## RESULTS

### Expression of E6 oncoprotein derived from HPV16 induces ECAC

**Fig 1:**
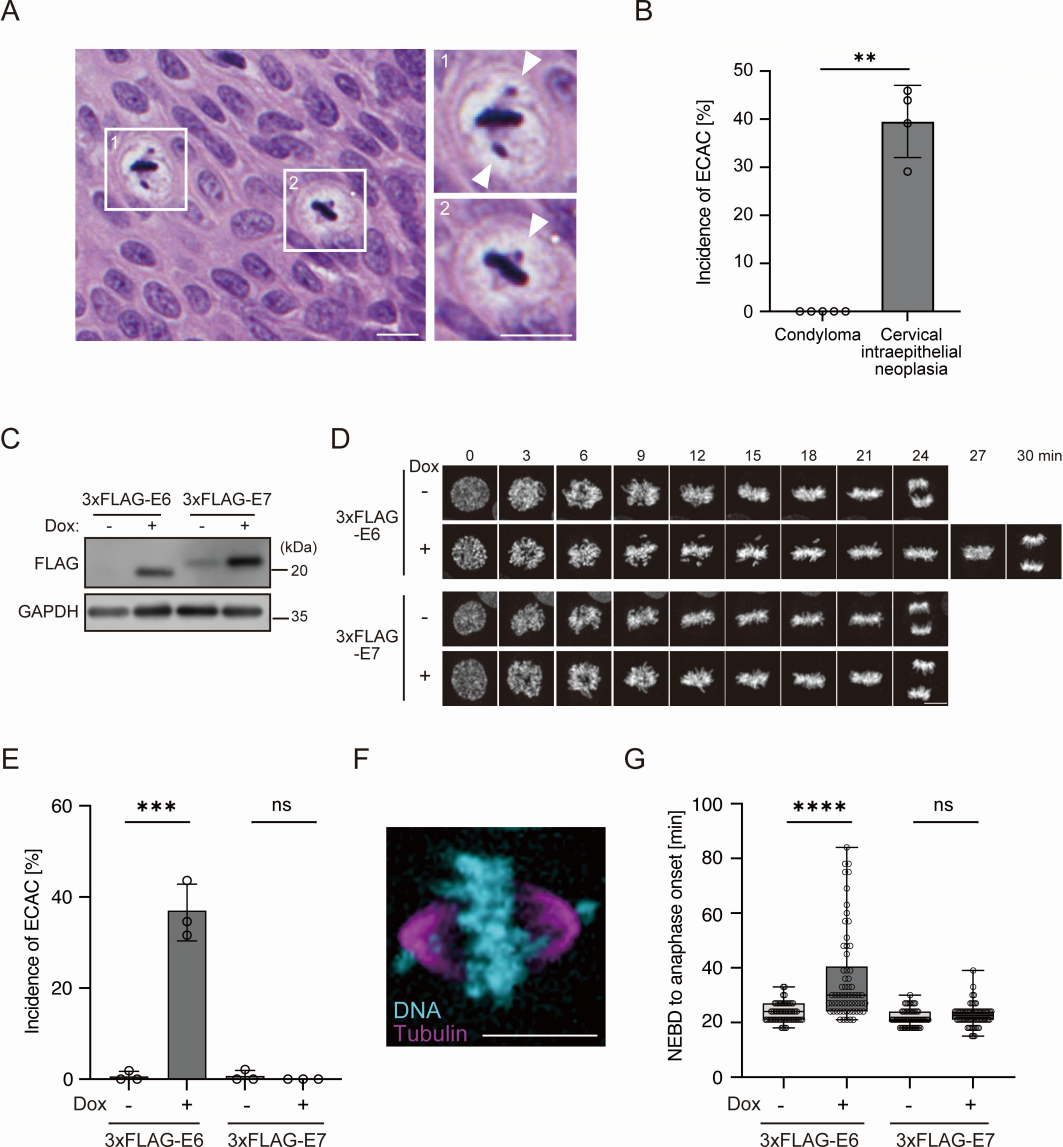
Ectopic chromosomes around centrosomes (ECACs) are observed in HPV16-positive cervical dysplastic lesions and recapitulated by HPV16 E6 expression in human RPE1 cells. (**A**) Representative H&E-stained cervical biopsies from HPV16-positive cervical intraepithelial neoplasia showing mitotic cells with ECACs, defined as chromosomes abnormally retained around centrosomes rather than properly congressed to the metaphase plate. Left: low-magnification overview; right: higher-magnification view of the boxed region. Arrowheads indicate ECACs. Examples shown include (1) unaligned chromosomes at both spindle poles and (2) unaligned chromosomes at one spindle pole. Scale bars, 10 μm. (**B**) ECAC frequency in each case, calculated as the percentage of mitotic figures exhibiting ECAC among all mitoses examined in that case. Each dot represents one case (low-risk HPV11-positive condyloma, *n* = 5; high-risk HPV16-positive cervical intraepithelial neoplasia, *n* = 4). For HPV16 cases, ≥23 mitotic figures were examined per case, whereas only 2–4 mitotic figures were available per HPV11 case. Bars indicate mean ± SD. *P* values were calculated using an unpaired two-tailed *t*-test with Welch’s correction. **P < 0.01. (**C**) Doxycycline (Dox)-inducible expression of 3xFLAG-E6 or 3xFLAG-E7 in non-transformed RPE1 cells. GAPDH served as a loading control. (**D**) Representative live-cell images of chromosomes in cells expressing 3xFLAG-E6 or 3xFLAG-E7. Chromosomes were visualized using SiR-DNA and imaged every 3 min. Elapsed times (min) after nuclear envelope breakdown (NEBD) are shown. Scale bar, 10 μm. (**E**) Incidence of ECAC in 3xFLAG-E6- or 3xFLAG-E7-expressing cells. ECAC was defined as the retention of one or more chromosomes near spindle poles for more than two consecutive frames (>3 min) without aligning at the metaphase plate. Each data point represents the incidence of ECAC calculated from an independent experiment (≥20 metaphase cells analyzed per experiment), and bars indicate mean ± SD (*n* = 3). *P* values were calculated using an unpaired two-tailed Student’s *t*-test. ns, not significant; ***P < 0.001. (**F**) Representative fluorescence images showing chromosomes persisting near centrosomes in 3×FLAG-E6-expressing cells. Chromosomes and microtubules were labeled with SiR-DNA and SiR-tubulin, respectively. Scale bar, 10 μm. (**G**) Box plots showing the duration from NEBD to anaphase onset in cells expressing 3xFLAG-E6 or 3xFLAG-E7. ≥62 cells were analyzed per condition, and each dot represents one cell. *P* values were calculated using the Mann-Whitney test: ns, not significant; ****P < 0.0001.

ECAC has been described as a characteristic mitotic abnormality in high-risk HPV-positive cervical dysplasia, in which chromosomes fail to align at the metaphase plate and instead remain near the centrosomes (Fig. 1A and B) (12). In histological examinations, ECAC was detected in approximately 40% of mitotic figures in cervical dysplasia, whereas it was not observed in the condyloma cases examined, a low-risk HPV-associated lesion (Fig. 1B). To determine how ECAC is induced in HPV-positive cells, we focused on the high-risk HPV16 oncoproteins E6 and E7, which are highly associated with cervical carcinogenesis (14, 15). These proteins deregulate cell cycle control by inactivating key tumor suppressors: E6 induces p53 degradation (16–18), while E7 inactivates RB (19, 20), thereby promoting carcinogenesis. We, therefore, reasoned that comparing the mitotic phenotypes of E6- and E7-expressing cells would clarify which oncoprotein is involved in the emergence of ECAC.

We first established non-transformed hTERT-immortalized RPE1 cell lines that express 3xFLAG-tagged E6 or 3xFLAG-tagged E7 in a doxycycline (Dox)-inducible manner (Fig. 1C). Live-cell imaging revealed a high frequency of chromosome congression defects in E6-expressing cells, whereas such defects were barely observed in E7-expressing cells (39.7% and 1.2%, respectively; Fig. 1D and E). Chromosomes that failed to align at the metaphase plate remained close to the spindle poles (Fig. 1F). This phenotype in E6-expressing cells was highly reminiscent of ECAC observed in cervical lesions (Fig. 1A and B) and is hereafter referred to as ECAC. Moreover, E6 expression significantly prolonged mitotic progression from nuclear envelope breakdown (NEBD) to anaphase onset (Fig. 1G), consistent with previous observations (21). These observations indicate that E6 expression perturbs chromosome congression, thereby inducing ECAC.

### Destabilization of p53 does not induce ECAC

HPV16 E6 is known to form a complex with the ubiquitin E3 ligase E6AP/UBE3A, leading to the degradation of various proteins through ubiquitin-dependent proteolysis. Among these, p53 is a relevant degradation target, and loss of p53 plays a significant role in HPV-associated carcinogenesis (22). Notably, p53 has been reported to localize to mitotic centrosomes and to contribute to spindle-pole integrity (23), raising the possibility that p53 reduction might contribute to ECAC.

**Fig 2:**
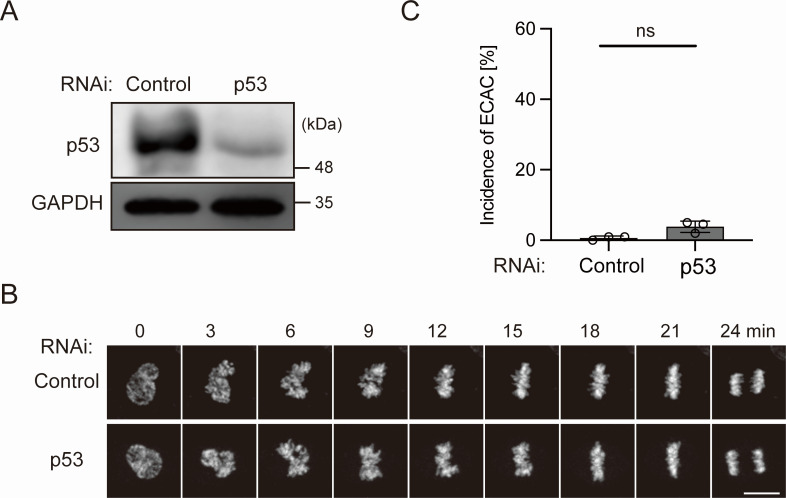
Reduction of p53 does not induce ECAC. (**A**) Western blot showing p53 depletion by RNA interference. GAPDH served as a loading control. (**B**) Representative live-cell images of chromosomes in p53-depleted cells. Chromosomes were visualized using SiR-DNA and imaged every 3 min. Elapsed time (min) after NEBD is shown. Scale bar, 10 μm. (**C**) Incidence of ECAC in p53-depleted cells. Each data point represents the incidence of ECAC calculated from an independent experiment (≥21 metaphase cells analyzed per experiment), and bars indicate mean ± SD (*n* = 3). *P* values were calculated using an unpaired two-tailed Student’s *t*-test. ns, not significant.

To test whether p53 loss leads to ECAC, we knocked down p53 using RNA interference in RPE1 cells and analyzed mitotic chromosome behavior (Fig. 2A). However, live-cell imaging revealed that p53 depletion barely influenced chromosome congression (Fig. 2B and C). Thus, p53 reduction alone does not account for ECAC.

### Degradation of CENP-E triggered by E6 expression induces ECAC

**Fig 3:**
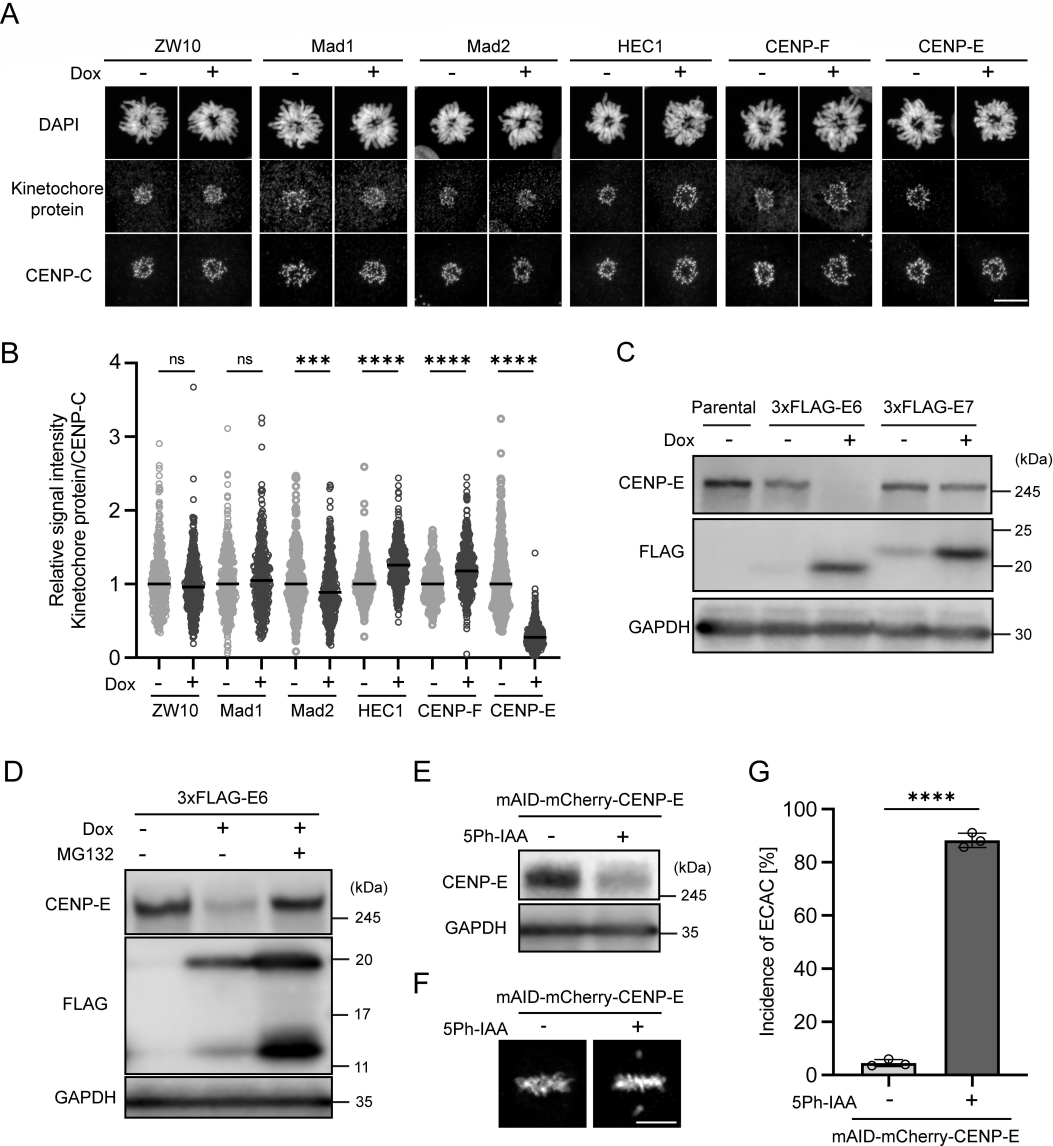
Degradation of CENP-E triggered by E6 expression induces ECAC. (**A**) Immunofluorescence images showing localization of kinetochore proteins in 3xFLAG-E6-expressing cells at prometaphase after Dox induction. DNA was stained with DAPI. Scale bar, 10 μm. (**B**) Quantification of kinetochore protein signals normalized to CENP-C intensity in prometaphase cells. ≥290 kinetochores from ≥8 cells per condition were analyzed. Each dot represents one kinetochore. *P* values were calculated using the Mann-Whitney test: ns, not significant; ***P < 0.001, ****P < 0.0001. (**C**) Western blot showing reduced CENP-E levels in 3xFLAG-E6-expressing cells, but not 3xFLAG-E7. GAPDH served as a loading control. (**D**) Western blot showing restoration of CENP-E levels in 3xFLAG-E6-expressing cells following MG132 treatment for 4 h. (**E**) Western blot showing depletion of mAID-mCherry-CENP-E after treatment with 5Ph-IAA for 2 h. (**F**) Representative live-cell images of metaphase cells with or without 5Ph-IAA treatment. Chromosomes were labeled with SiR-DNA. Scale bar, 10 μm. (**G**) Incidence of ECAC in mAID-mCherry-CENP-E cells treated with or without 5Ph-IAA. Each data point represents the incidence of ECAC calculated from an independent experiment (≥21 metaphase cells analyzed per experiment), and bars indicate mean ± SD (*n* = 3). *P* values were calculated using an unpaired two-tailed Student’s *t*-test. ****P < 0.0001.

Because ECAC already emerges in prometaphase, when kinetochore-microtubule (KT-MT) attachments occur (Fig. 1D), we reasoned that E6 might impair kinetochore proteins required for chromosome congression. To address this possibility, we analyzed components of fibrous corona (ZW10, Mad1, Mad2, CENP-F, and CENP-E), which mediate the initial lateral KT-MT interactions, and of outer kinetochore (HEC1), which mediates the end-on attachments (24). Among these, CENP-E, a mitotic kinesin known to be involved in chromosome congression, showed the most substantial reduction at kinetochores, becoming nearly undetectable in E6-expressing cells (Fig. 3A and B).

This impaired kinetochore localization of CENP-E was due to a reduction in total CENP-E protein levels, specifically induced by E6 but not E7 expression (Fig. 3C). Because HPV E6 is known to direct ubiquitin-mediated degradation of its targets through the ubiquitin ligase E6AP (25), we tested whether CENP-E reduction involves the proteasome pathway. Treatment of cells with a proteasome inhibitor MG132 restored CENP-E levels to those of control cells, suggesting that E6 promotes proteasome-dependent degradation of CENP-E (Fig. 3D).

To be able to directly test whether loss of CENP-E causes ECAC, we endogenously tagged CENP-E with auxin-inducible degron 2 (AID2) and acutely depleted CENP-E (Fig. 3E). As anticipated, CENP-E degradation phenocopied the congression defects observed in E6-expressing cells, resulting in a high incidence of ECAC (89.6%; Fig. 3F and G). These results strongly suggest that E6-mediated CENP-E destabilization contributes to ECAC in the context of HPV16 E6 expression.

### High-risk HPV E6 proteins broadly induce ECAC and CENP-E destabilization

**Fig 4:**
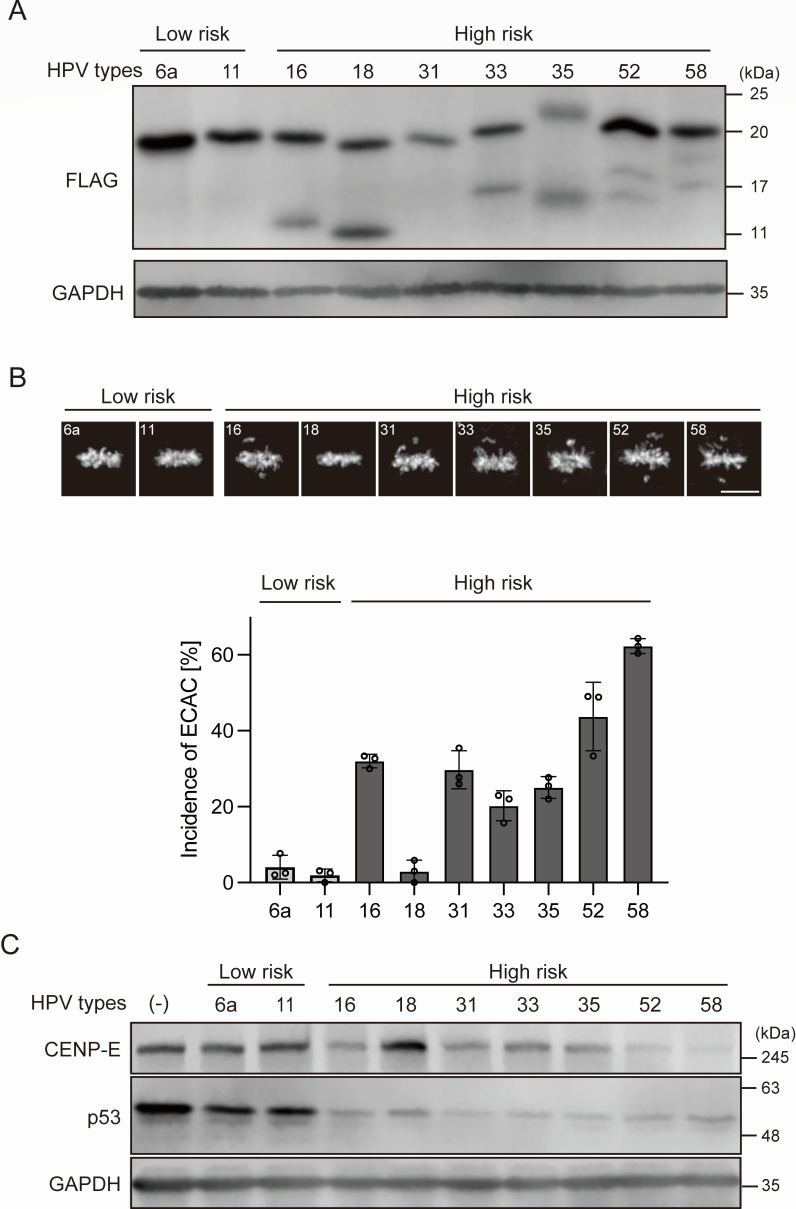
E6 variants from diverse HPV genotypes differentially destabilize CENP-E and induce ECAC. (**A**) Western blot showing the expression of 3xFLAG-tagged E6 variants derived from different HPV genotypes after Dox induction. MG132 was added to inhibit proteasome-mediated degradation of E6 targets. GAPDH served as a loading control. (**B**) (Top) Representative live-cell images of chromosomes in RPE1 cells expressing the indicated 3xFLAG-E6 variants. DNA was stained with SiR-DNA. The HPV genotype is indicated in the top left of each panel. Scale bar, 10 μm. (Bottom) Incidence of ECAC in RPE1 cells expressing the corresponding 3xFLAG-E6 variants. Each data point represents the incidence of ECAC calculated from an independent experiment (≥32 metaphase cells analyzed per experiment), and bars indicate mean ± SD (*n* = 3). (**C**) Western blot showing p53 and CENP-E levels in cells expressing each 3xFLAG-E6 variant.

Our analyses revealed that HPV16 E6 induces ECAC in non-transformed RPE1 cells. ECAC has also been reported in cervical dysplasia lesions harboring high-risk HPV genotypes, including HPV16 (12), underscoring its pathological relevance in cervical premalignancy. These observations prompted us to examine whether ECAC induction represents a shared property of E6 proteins from high-risk HPV types, or whether it varies among viral genotypes. To address this, we generated RPE1 cell lines that inducibly express 3 × FLAG-tagged E6 variants from epidemiologically defined low-risk (HPV6a, HPV11) and high-risk (HPV16, 18, 31, 33, 35, 52, 58) genotypes at comparable levels upon Dox addition (Fig. 4A).

In live-cell imaging analysis, E6 variants from low-risk HPV genotypes barely induced ECAC (4.9% and 1.3%). In contrast, E6 variants from several high-risk HPV genotypes (HPV16, 31, 33, 35, 52, 58) induced ECAC at markedly higher frequencies (31.3%–61.3%; Fig. 4B), suggesting that ECAC is a characteristic mitotic phenotype of high-risk E6 expression. Consistent with this ECAC-inducing activity, CENP-E protein levels were proportionally reduced in cells expressing these high-risk E6 variants (Fig. 4C). While HPV18 E6 induced ECAC only weakly (2.9%) and did not destabilize CENP-E, this behavior was distinct from other high-risk types. Although p53 reduction was commonly observed across all high-risk HPV E6 variants, as previously reported (26), this did not correlate with ECAC induction. Therefore, ECAC induction is associated with high-risk HPV genotypes and closely linked to CENP-E destabilization, with HPV18 representing a notable exception.

### CENP-E degradation is the primary cause of E6-induced ECAC

**Fig 5:**
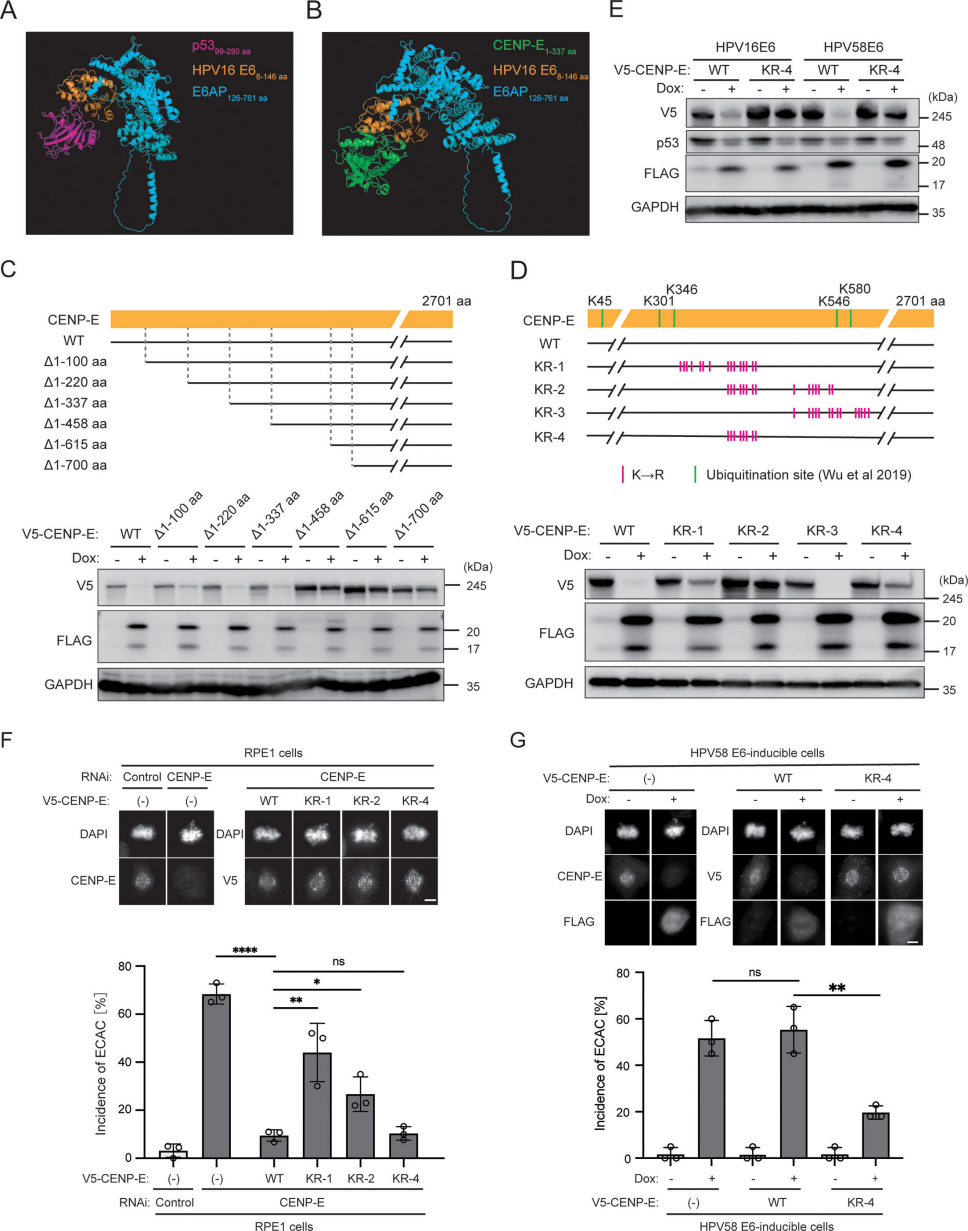
Stabilization of CENP-E prevents ECAC caused by E6. (**A**) AlphaFold3 (AF3) prediction of the trimeric p53-HPV16 E6-E6AP complex. The predicted structure closely matched the reported arrangement (RMSD = 0.956 Å), supporting the reliability of the AF3 model. Proteins were modeled using the following residue ranges: p53 (99–290 aa, magenta), HPV16 E6 (8–146 aa, orange), and E6AP (126–761 aa, cyan). (**B**) AF3 prediction of the CENP-E-HPV16 E6-E6AP complex. The N-terminal region of CENP-E (1–337 aa, green) was predicted to lie adjacent to HPV16 E6, analogous to the p53-E6-E6AP arrangement. The Predicted Aligned Error (PAE) plot showed low positional uncertainty for the CENP-E (1–337 aa) region relative to the E6-E6AP complex, whereas other regions exhibited higher uncertainty. HPV16 E6 and E6AP were modeled using the same residue ranges as in panel A. (**C**) Schematic representation of CENP-E N-terminal truncation mutants generated by incremental deletion of approximately 100 amino acids (top). Western blot showing the levels of V5-tagged CENP-E truncation mutants in cells with Dox-inducible expression of 3xFLAG-HPV58 E6 (bottom). GAPDH served as a loading control. (**D**) Schematic representation of CENP-E KR mutants (lysine-to-arginine substitutions), with substituted lysine residues indicated by pink lines and previously reported ubiquitination sites indicated by green lines (28) (top). Western blot showing the levels of V5-tagged CENP-E WT and KR mutants in cells expressing 3xFLAG-HPV58 E6 with or without Dox induction (bottom). GAPDH served as a loading control. (**E**) KR-4 mutant is also resistant to degradation by HPV16 E6. Western blot showing the levels of V5-tagged CENP-E WT and KR-4 in cells expressing 3xFLAG-HPV16 E6 or 3xFLAG-HPV58 E6, with or without Dox induction. p53 is shown as a positive control for E6 activity. GAPDH served as a loading control. (**F**) Immunofluorescence images showing endogenous CENP-E after control or CENP-E RNAi treatment, and V5-tagged CENP-E WT, KR-1, KR-2, or KR-4 in CENP-E-depleted cells. DNA was stained with DAPI. Scale bar, 10 μm (top). Incidence of ECAC in cells expressing the indicated V5-tagged CENP-E constructs after depletion of endogenous CENP-E by RNAi. Cells treated with control RNAi without ectopic V5-CENP-E expression served as a baseline control, whereas cells depleted of endogenous CENP-E without ectopic V5-CENP-E expression served as a positive control. Each data point represents the incidence of ECAC calculated from an independent experiment (≥20 metaphase cells analyzed per experiment), and bars indicate mean ± SD (*n* = 3). Statistical analysis was performed using one-way ANOVA followed by Dunnett’s multiple-comparisons test. ns, not significant; *P < 0.05; **P < 0.01; ****P < 0.0001 (bottom). (**G**) Immunofluorescence images showing endogenous CENP-E or V5-tagged CENP-E WT or KR-4 in HPV58 E6-inducible cells with or without Dox induction. DNA was stained with DAPI. Scale bar, 10 μm (top). Incidence of ECAC in HPV58 E6-inducible cells with endogenous CENP-E or expressing V5-tagged CENP-E WT or KR-4, with or without Dox induction. Each data point represents the incidence of ECAC calculated from an independent experiment (≥20 metaphase cells analyzed per experiment), and bars indicate mean ± SD (*n* = 3). *P* values were calculated using an unpaired two-tailed Student’s *t*-test. ns, not significant; **P < 0.01 (bottom).

In order to determine whether CENP-E degradation is required for ECAC, we next aimed to generate a degradation-resistant CENP-E mutant. AlphaFold3 modeling predicted that the N-terminal region of CENP-E may contact the E6/E6AP complex in a configuration reminiscent of the reported p53-E6-E6AP structure (Fig. 5A and B) (27), raising the possibility that this region targets CENP-E for E6-dependent degradation.

To narrow down the region responsible for this process, we examined a series of N-terminal truncation mutants (Fig. 5C). Using E6 derived from HPV58, which reduced CENP-E level most strongly among the variants tested (Fig. 4C), we found that degradation remained intact in the mutant deleted up to aa 337 (Δ1–337) and that it became largely resistant when deleting to aa 458 (Fig. 5C). Thus, the region spanning aa 338–458 contains sequences relevant for the degradation. Because this degradation is proteasome-dependent (Fig. 3D), we tested a series of lysine-to-arginine mutants (KR-1, -2, -3, and -4), in which clusters of lysines, within the broader 338–615 aa region, were all replaced with arginine to block ubiquitination, excluding lysines required to establish stable KT-MT attachments (Fig. 5D) (28). Upon expression of E6, we found that KR-1, KR-2, and KR-4 mutants showed resistance to degradation (Fig. 5D). The KR-4 mutant was also resistant to degradation by HPV16 E6, indicating that resistance is not limited to HPV58 and that multiple HPV types likely target the same region of CENP-E (Fig. 5E).

Next, to assess their functionality, these KR mutants were expressed in place of endogenous CENP-E. Among the mutants tested, the V5-tagged CENP-E KR-4 mutant was the only mutant that showed a level of suppression comparable to that of V5-tagged CENP-E wild type (WT) (Fig. 5F). These results indicate that the KR-4 mutant retains the ability to support proper chromosome alignment. Having non-degradable CENP-E in hand, we then evaluated ECAC frequency upon E6 expression in cells expressing V5-CENP-E KR-4. Under conditions in which E6 expression destabilized V5-CENP-E WT and induced ECAC, comparable levels of the V5-CENP-E KR-4 mutant remained localized at kinetochores and effectively prevented ECAC (Fig. 5G). These findings establish that E6-induced ECAC is primarily caused by the degradation of CENP-E.

### Relevance of the E6-CENP-E axis in HPV-positive cancer cells

**Fig 6:**
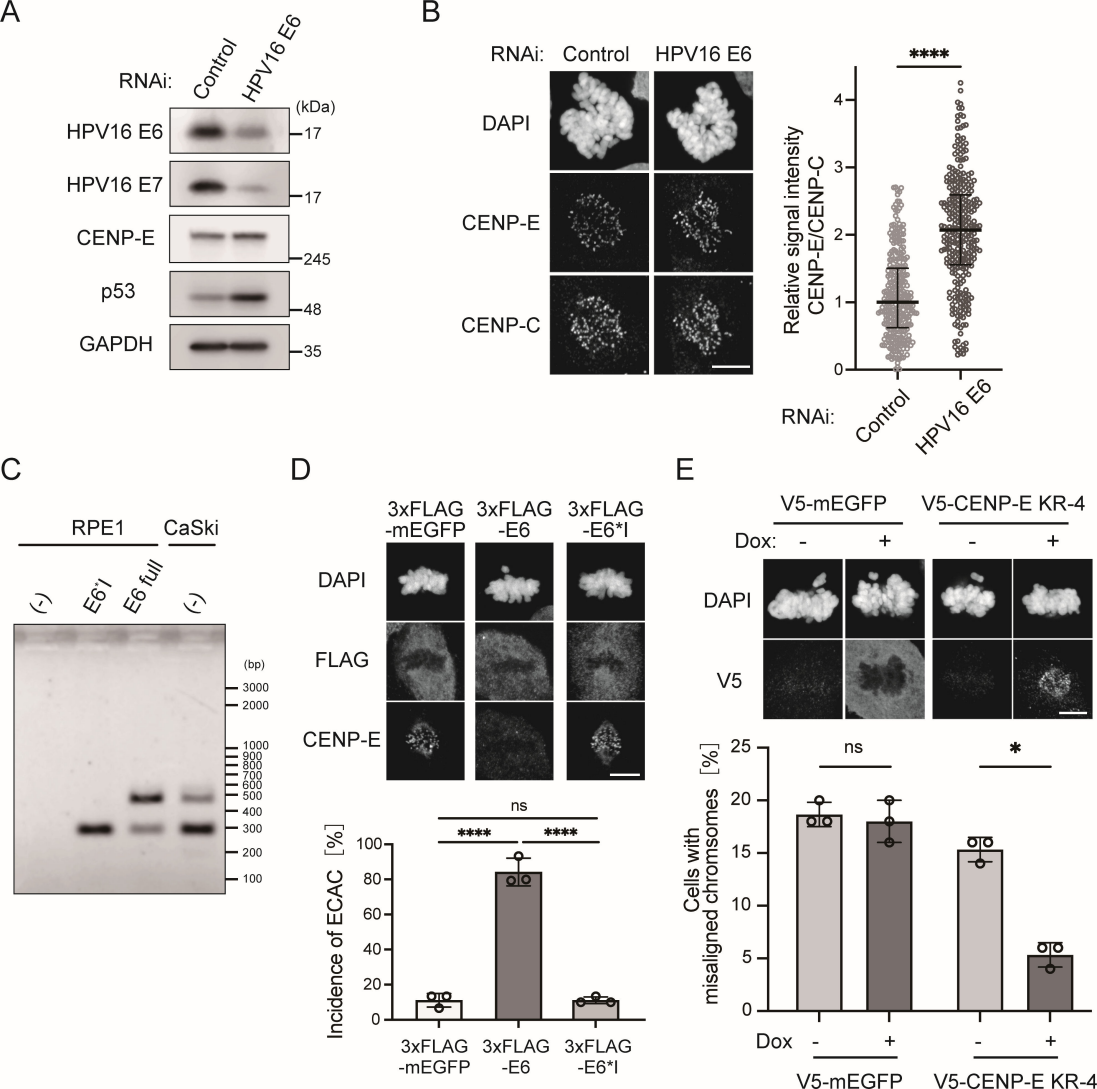
The E6-CENP-E axis remains functionally active in HPV-positive cancer cells. (**A**) Western blot showing the levels of HPV16 E6, HPV16 E7, p53, and CENP-E after RNAi-mediated depletion of E6 in CaSki cells. The siRNA targets a sequence shared by the full-length E6 transcript and the spliced transcript variant E6*I. Because E6 and E7 are derived from a common polycistronic transcript, E7 levels were also reduced. GAPDH served as a loading control. (**B**) Immunofluorescence images showing CENP-E and CENP-C localization in prometaphase CaSki cells treated with control or HPV16 E6 RNAi. DNA was stained with DAPI. Scale bar, 10 μm (left). Quantification of CENP-E signal intensity normalized to CENP-C intensity. 300 kinetochores from 10 cells per condition were analyzed. Each dot represents one kinetochore. *P* values were calculated using the Mann-Whitney test. ****P < 0.0001 (right). (**C**) Reverse transcription PCR analysis of E6 transcripts in doxycycline-inducible RPE1 cells expressing HPV16 E6*I or full-length HPV16 E6, and in CaSki cells, followed by agarose gel electrophoresis. (**D**) Representative immunofluorescence images showing CENP-E localization in mitotic RPE1 cells transiently expressing 3xFLAG-mEGFP, full-length 3xFLAG-HPV16 E6, or 3xFLAG-HPV16 E6*I. DNA was stained with DAPI. Scale bar, 10 μm (top). Incidence of ECAC in RPE1 cells transiently expressing 3xFLAG-mEGFP, full-length 3xFLAG-HPV16 E6, or 3xFLAG-HPV16 E6*I. Each data point represents the incidence of ECAC calculated from an independent experiment (30 metaphase cells analyzed per experiment), and bars indicate mean ± SD (*n* = 3). *P* values were calculated using one-way ANOVA followed by Tukey’s multiple-comparisons test. ns, not significant; ****P < 0.0001 (bottom). (**E**) Immunofluorescence images showing V5-mEGFP or V5-tagged CENP-E KR-4 in doxycycline-inducible CaSki cell lines with or without Dox induction. DNA was stained with DAPI. Scale bar, 10 μm (top). Incidence of cells with misaligned chromosomes in these cell lines, with or without Dox induction. Each data point represents the incidence of cells with misaligned chromosomes calculated from an independent experiment (50 metaphase cells analyzed per experiment), and bars indicate mean ± SD (*n* = 3). *P* values were calculated using an unpaired two-tailed Student’s *t*-test. ns, not significant; *P < 0.05 (bottom).

The fact that ECAC is typically seen in dysplasia but not in the subsequent cancers (12) led us to ask whether the E6-CENP-E axis also operates in HPV-positive cancer cells. To address this, we depleted endogenous E6 in CaSki cells, an HPV16-positive cancer cell line. We found that E6 depletion resulted in stabilization of both p53 and CENP-E (Fig. 6A), and a twofold increase in the level of kinetochore-associated CENP-E (Fig. 6B). These results indicate that endogenous E6 contributes to the downregulation of both p53 and CENP-E in CaSki cells.

Because E6-dependent destabilization of CENP-E appeared more modest in CaSki cells than in the RPE1 model, we next examined the E6 transcript composition in CaSki cells. High-risk HPVs are known to express both full-length E6 transcripts and spliced isoforms such as E6*I, and E6*I is often the predominant species in HPV-positive cells (29, 30). Consistent with this, RT-PCR analysis showed that CaSki cells predominantly expressed E6*I, whereas the HPV16 E6-expressing RPE1 cells used in this study predominantly expressed full-length E6 (Fig. 6C). Unlike full-length E6, E6*I failed to destabilize CENP-E or induce ECAC (Fig. 6D). These results suggest that the modest reduction of CENP-E in CaSki cells is likely driven by the full-length E6 still present despite the predominance of E6*I. Indeed, the expression of the degradation-resistant CENP-E KR4 mutant in CaSki cells significantly reduced chromosome misalignment (Fig. 6E). These results indicate that the E6-CENP-E axis remains functionally active in HPV-positive cancer cells.

### Sustained ECAC promotes the emergence of aneuploid cell populations

**Fig 7:**
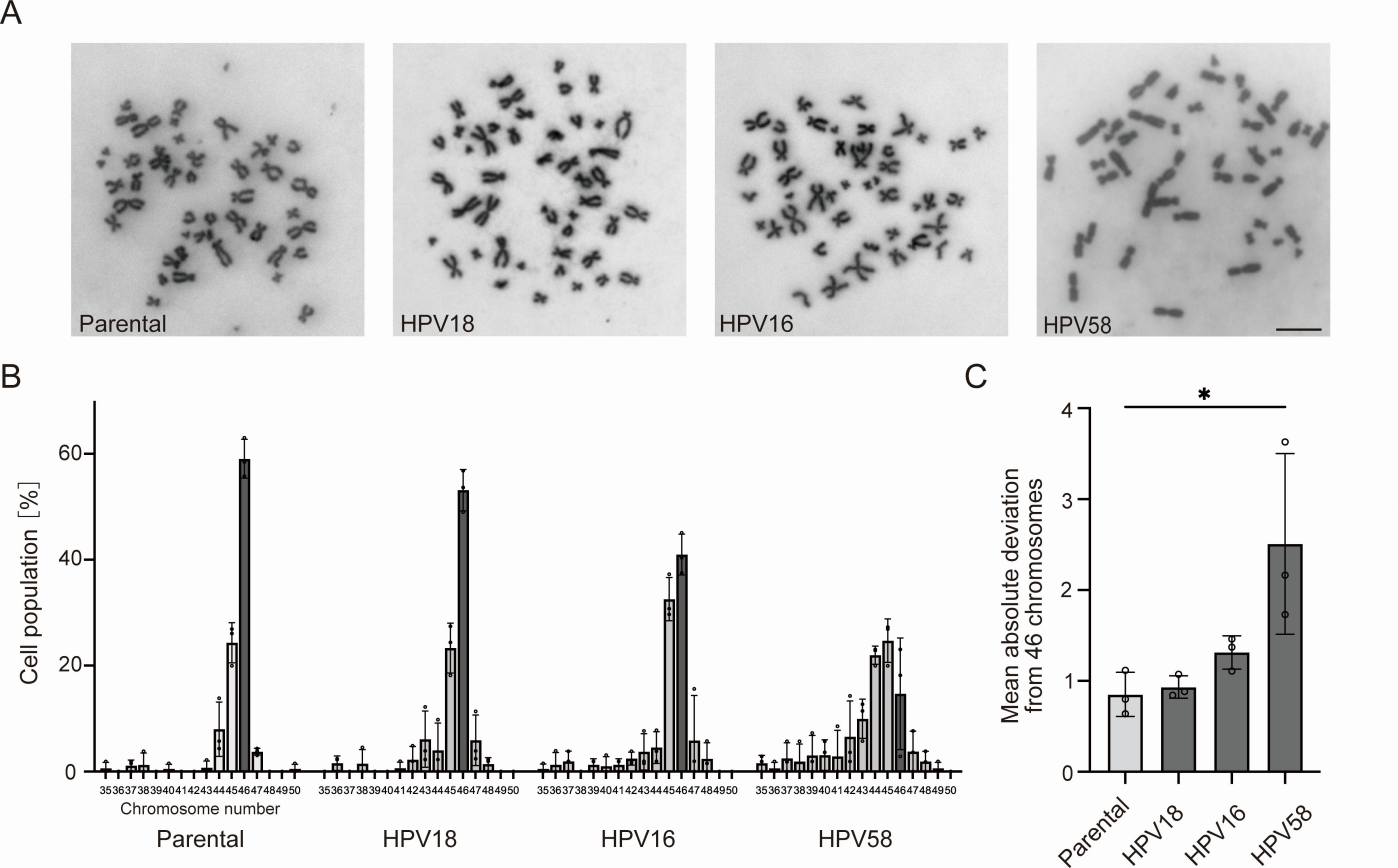
ECAC promotes chromosome number alterations in E6-expressing cell. (**A**) Representative chromosome-spread images from parental RPE1 cells and RPE1 cells expressing HPV16, HPV18, or HPV58 E6 after long-term doxycycline induction. Scale bar, 10 μm. (**B**) Distribution of chromosome numbers per cell in parental RPE1 cells and RPE1 cells expressing HPV16, HPV18, or HPV58 E6. Data represent mean ± SD from three independent experiments, with ≥38 metaphase cells analyzed per experiment. Individual data points are shown as dots. (**C**) Chromosome number variability was quantified as the mean absolute deviation from the diploid chromosome number (46) for each biological replicate. Each data point represents an independent experiment, and bars indicate mean ± SD (*n* = 3). *P* values were calculated using the Kruskal-Wallis test followed by Dunn’s multiple-comparisons test against the parental condition. *P < 0.05.

Finally, to address whether sustained ECAC may cause an increase in aneuploid cell populations, we cultured RPE1 cells expressing HPV16, HPV18, or HPV58 for 1 month and assessed their chromosome numbers by metaphase spreads. In HPV18 E6-expressing cells, which do not exhibit ECAC, the modal chromosome number remained largely unchanged at 46, similar to parental cells. In contrast, HPV16 E6-expressing cells, in which ECAC frequently occurred, displayed a modest shift in the modal chromosome number to 45. Notably, HPV58 E6-expressing cells, which exhibited the strongest ECAC phenotype, showed a broader chromosome number distribution skewed toward chromosome loss (Fig. 7A and B). In a quantitative assessment for the extent of this redistribution, we found chromosome-number variability was significantly increased in HPV58 E6-expressing cells (Fig. 7C). An implication from these results is that ECAC contributes to the emergence of aneuploid cell populations through the acquisition of chromosomal instability.

## DISCUSSION

Our study places the E6-CENP-E axis in the context of cervical premalignancy, where ECAC is observed as a characteristic chromosome congression defect in high-risk dysplasia (Fig. 1), and addresses whether this defect might facilitate the acquisition of chromosomal instability. Our data show that E6 proteins across multiple high-risk HPV genotypes, not only HPV16, induce CENP-E degradation-dependent chromosome congression defects (Fig. 4). Restoration of CENP-E levels by the expression of a degradation-resistant mutant was sufficient to suppress ECAC, establishing CENP-E destabilization as the primary causal event underlying this phenotype. Through the truncation and lysine-cluster mutagenesis analyses, these results further define the CENP-E region required for E6-dependent degradation (Fig. 5). In addition, although ECAC is most readily observed in premalignant cervical lesions, the experiments with CaSki cells suggest that the underlying E6-CENP-E axis remains active, at least in part, in HPV-positive cancer cells (Fig. 6). Importantly, the persistence of ECAC drove the emergence of aneuploid cell populations in non-transformed diploid cells (Fig. 7). These findings establish ECAC as a cytological consequence of E6-dependent CENP-E destabilization in the context of cervical dysplasia and support the idea that ECAC constitutes an early step toward chromosomal instability.

How does CENP-E reduction mechanistically link ECAC to chromosome-number alterations? CENP-E transports pole-proximal chromosomes toward the spindle equator and, during this process, promotes the maturation of lateral kinetochore-microtubule attachments into stable end-on attachments, a conversion step essential for accurate chromosome segregation (24, 31). Consistent with this critical role, even partial reduction of CENP-E causes chromosome misalignment and subsequent missegregation, resulting in aneuploidy (32). In this context, the phenotypes observed in E6-expressing cells, including persistent polar chromosomes, delayed congression, and altered chromosome number, can be attributed to reduced CENP-E levels (Fig. 1 and 7). These observations indicate that ECAC reflects a sustained failure in kinetochore-microtubule attachment maturation, predisposing cells to chromosome missegregation and chromosome-number alterations.

The redistributed chromosome-number patterns observed in our long-term cultures (Fig. 7) likely reflect both the initial missegregation events caused by ECAC and subsequent selection driven by the fitness consequences of specific karyotypic changes. Consistent with this idea, in HPV58-expressing cells the distribution converged toward lower chromosome numbers, suggesting that cells harboring chromosome gains may be selectively disadvantaged under these culture conditions. An important question for future studies is whether any specific chromosomes are preferentially involved in ECAC, or whether ECAC affects chromosomes in an unbiased fashion and the resulting karyotypes are subsequently shaped by the selection.

The E6 expression from multiple high-risk HPV genotypes (HPV16, 31, 33, 35, 52, and 58) induced ECAC at high frequencies (Fig. 4). These HPV genotypes are strongly predisposed to progress from dysplasia to squamous cell carcinoma (33). This tight correlation raises the possibility that ECAC may represent a cytological event linking dysplasia and carcinoma. Notably, HPV18, whose E6 neither degrades CENP-E nor induces ECAC, represents an exceptional high-risk genotype that does not follow the dysplasia-carcinoma sequence but is instead more frequently associated with adenocarcinoma *in situ* and invasive adenocarcinoma (33). These findings suggest that HPV18 E6 targets a distinct set of molecules compared with other high-risk types, potentially directing infected cells toward an alternative route of malignant transformation.

High-risk HPV E6 has long been recognized as an oncoprotein that promotes malignant transformation through p53 degradation. Our findings reveal an additional mechanism by which E6 can compromise genome maintenance: it destabilizes CENP-E, thereby impairing mitotic fidelity and promoting the accumulation of aneuploid cells (Fig. 7). Given that p53 loss increases cellular tolerance to aneuploidy, the combined effects of E6-mediated p53 inactivation and CENP-E destabilization provide a plausible synergistic mechanism that could accelerate chromosomal instability during carcinoma initiation. Together with recent observations of related mitotic defects in HPV-associated head and neck cancers (13), these findings suggest that chromosome congression defects represent a broader pathological hallmark of high-risk HPV infection and may constitute a previously underappreciated route of carcinogenesis. At the same time, reduced CENP-E activity may also create a vulnerability under conditions of additional genomic stress. Consistent with this idea, mice with reduced CENP-E levels (CENP-E+/−) exhibit suppression of tumor formation when exposed to DNA-damaging stress due to excessive chromosomal instability (32). By analogy, ECAC-harboring cells may likewise exhibit such vulnerability, suggesting a potential therapeutic opportunity.

## MATERIALS AND METHODS

### Cell culture, cell lines, and lentivirus

hTERT-RPE1 cells (ATCC CRL-4000) were cultured in high-glucose DMEM supplemented with 10% fetal bovine serum (FBS) and 10 mM HEPES (pH 7.5) at 37°C in a humidified atmosphere containing 5% CO₂. CaSki cells (JCRB Cell Bank, IFO50007) were cultured in RPMI 1640 supplemented with 10% fetal bovine serum (FBS) under the same conditions. Cells were routinely tested and confirmed to be free of mycoplasma contamination.

TetON RPE1 cells were generated by introducing the TetR gene into a puromycin-resistance-deleted hTERT-RPE1 cell line previously established (34) using the PiggyBac transposon system. Transfection was performed with the Neon transfection system (1350 V, 20 ms, 2 pulses; Thermo Fisher Scientific). Stable cell populations were selected with blasticidin (50 µg/mL), and clonal cell lines were isolated by limiting dilution.

Doxycycline-inducible HPV16 E6, E6*I, or E7 expression cell lines were generated by inserting the E6, E6*I, or E7 coding sequence into a PiggyBac vector containing an N-terminal 3×FLAG tag and co-transfecting it with a PiggyBac transposase plasmid into TetON RPE1 cells using the same Neon transfection conditions. Puromycin selection (1.5 µg/mL) and single-cell cloning by limiting dilution were employed to obtain clonal inducible cell lines. Transgene expression was induced by treating cells with 2 µg/mL doxycycline for 24 h.

To establish cell populations expressing various HPV E6 variants (types 6a, 11, 16, 18, 31, 33, 35, 52, and 58), the corresponding coding sequences were cloned into the pSLQ lentiviral vector containing an N-terminal 3 × FLAG tag. Lentiviral particles were generated by transfecting the vectors together with a packaging mix (ViraPower, Thermo Fisher Scientific) into HEK293T cells using polyethyleneimine (PEI). The culture supernatant was harvested 72 h after transfection and concentrated using Lenti-X Concentrator (Takara). The concentrated lentivirus was applied to TetON RPE1 cells for 48 h, after which puromycin selection (1.5 µg/mL) was initiated. To obtain cell populations with comparable E6 expression levels, cells expressing lower E6 levels were subjected to an additional round of infection.

To conditionally deplete CENP-E, the auxin-inducible degron 2 (AID2) system was employed to enable rapid degradation upon addition of 5Ph-IAA (35). The mAID-mCherry fusion tag was inserted at the N-terminus of endogenous CENP-E on both alleles using the CRISPR/Cas9 system. Stable cell lines were selected with blasticidin (50 µg/mL), and clonal lines were isolated by limiting dilution. Correct allele replacement and degradation kinetics were validated by genomic PCR, immunoblotting, and auxin-induced degradation assays. To induce degradation of AID-tagged CENP-E, cells were treated with 1 µM 5Ph-IAA for 2 h.

To identify potential ubiquitination sites, clusters of lysine residues within the coiled-coil region of CENP-E were substituted with arginine. Four lysine-to-arginine (KR) mutant groups were generated, in which the following lysines were replaced: KR-1 (K350, K356, K357, K373, K383, K388, K393, K414, K416, K418, K427, K430, K432, K449, K452); KR-2 (K414, K416, K418, K427, K430, K432, K449, K452, K489, K521, K527, K529, K531, K543, K545); KR-3 (K489, K521, K527, K529, K531, K543, K545, K560, K564, K587, K592, K593, K602, K607); KR-4 (K414, K416, K418, K427, K430, K432, K449, K452). These CENP-E mutants were cloned into a PiggyBac vector containing a V5 tag and introduced into doxycycline-inducible HPV16- or HPV58-E6-expressing RPE1 cells together with a PiggyBac transposase plasmid to enable genomic integration. Cells resistant to G418 (3 mg/mL) were selected.

Doxycycline-inducible CaSki cell lines expressing V5-mEGFP or V5-CENP-E KR-4 were generated by inserting the mEGFP or CENP-E KR-4 coding sequence into a PiggyBac vector containing an N-terminal V5 tag and the TetON3G cassette, and co-transfecting it together with a PiggyBac transposase plasmid into CaSki cells using the Neon transfection system (1100 V, 20 ms, 2 pulses). Stable cell populations were selected with puromycin (1.5 µg/mL). Transgene expression was induced by treating cells with 2 µg/mL doxycycline for 24 h.

### RNA interference

RNA interference was performed by transfecting cells with 10 nM siRNAs using Lipofectamine RNAiMAX (Thermo Fisher Scientific) according to the manufacturer’s instructions. Cells were analyzed 48 h after transfection for p53 knockdown and 24 h after transfection for CENP-E knockdown. The target sequence for p53 was 5′-CCAUCCACUACAACUACAUGUGUAA-3′ (Stealth RNA, Thermo Fisher Scientific). The siRNA targeting the 3′ untranslated region (UTR) of CENP-E was 5′-GUAUACUUUUAAAAGUUGAAUUGU-3′ (IDT). The target sequence for HPV16 E6 was 5′-GACCGGUCGAUGUAUGUCUUG-3′ (Thermo Fisher Scientific), which is shared by full-length E6 and E6*I. As a control, MISSION siRNA Universal Negative Control #1 (Sigma-Aldrich) was used.

### Immunofluorescence microscopy

Cells were fixed with 2% paraformaldehyde (PFA) for 10 min, permeabilized with 0.2% Triton X-100 for 10 min, and blocked with 3% BSA for 10 min. Primary antibodies were incubated for 2 h at room temperature: anti-FLAG (1:10,000; F1804, Sigma-Aldrich), anti-V5 (1:1,000; 13,202, Cell Signaling Technology), anti-ZW10 (1:1,000; 24561-1-AP, Proteintech), anti-Mad1 (1:1,000; sc-47746, Santa Cruz), anti-Mad2 (1:1,000; 610,678, BD Transduction Laboratories), anti-HEC1 (1:5,000; ab3613, Abcam), anti-CENP-F (1:3,000; NB500-101, Novus Biologicals), anti-CENP-E (1:1,000; ab5093 or ab133583, Abcam), and anti-CENP-C (1:5,000; PD030, MBL). Secondary antibodies conjugated with Alexa Fluor 488, 568, or 647 (anti-mouse, anti-rabbit, or anti-guinea pig IgG; all at 1:500) were incubated for 1 h at room temperature together with DAPI (10 ng/mL) for DNA staining. Coverslips were then mounted using ProLong Gold antifade mounting reagent (Invitrogen). Images were acquired using a Zeiss Axio Imager M1 fluorescence microscope equipped with a Prime BSI camera (Teledyne Photometrics) or a confocal laser scanning microscope (LSM 880; Zeiss).

Quantification of kinetochore protein intensities was performed as previously described (36), with minor modifications. ROI-based measurements (six-pixel diameter) were obtained from maximum-intensity projection images acquired using the LSM 880 system, with CENP-C defining kinetochore positions. Image processing and intensity measurements were performed using Fiji (ImageJ). Background-subtracted intensities were expressed as the ratio of kinetochore protein to CENP-C.

### Immunoblotting

Cells were lysed in Omnicleave buffer (1 mM MgCl₂, 0.1% Triton X-100, and 0.1% Omnicleave endonuclease; OC7850K, Cambio) for 5 min at room temperature to digest DNA. Lysates were then mixed with SDS sample buffer and boiled for 5 min. Proteins were separated by SDS-PAGE and transferred onto PVDF membranes (Immobilon-P, Millipore).

Primary antibodies were incubated overnight at 4°C: anti-FLAG (1:5,000; F1804, Sigma-Aldrich), anti-V5 (1:1,000; 13,202, Cell Signaling Technology), anti-p53 (1:5,000; sc-47698, Santa Cruz), anti-CENP-E (1:1,000; ab5093, Abcam), anti-GAPDH (1:20,000; #2118, Cell Signaling Technology), anti-HPV16 E6 (1:5,000; GTX132686, GeneTex), and anti-HPV16 E7 (1:1,000; sc-6981, Santa Cruz Biotechnology). HRP-conjugated secondary antibodies were used at 1:1,000 and incubated for 1 h at room temperature. Chemiluminescent signals were developed using a luminol-coumaric acid substrate (Sigma) and detected with an Odyssey imaging system (LI-COR).

### Chromosome spreads

Mitotic cells were collected by shake-off after 2.5 h of synchronization with 200 ng/mL colcemid (AdipoGen). Cells were incubated in a hypotonic buffer (PBS:H₂O = 3:7) for 5 min at room temperature and then fixed with freshly prepared Carnoy’s fixative (methanol:acetic acid = 3:1). Fixed cells were dropped onto glass slides and air-dried. Chromosomes were stained with 5% Giemsa solution for 20 min, rinsed three times with distilled water, air-dried, and mounted with Entellan mounting medium (Sigma-Aldrich).

### Live-cell imaging analysis

Live-cell imaging of cell cycle progression was performed using a CQ1 confocal quantitative image cytometer (Yokogawa). Cells were seeded into 24-well glass-bottom plates (Iwaki) and cultured overnight. DNA was stained with 100 nM SiR-DNA (Cytoskeleton, Inc.) for 1 h prior to imaging. For tubulin labeling, cells were treated with 10 nM SiR-Tubulin (Cytoskeleton, Inc.) for 1 h. Time-lapse images were acquired every 3 min using a UPLXAPO 40 × objective lens. Maximum-intensity projection images generated from seven Z-sections spaced 9 µm apart were analyzed using Fiji (ImageJ). For CENP-E degradation experiments, mAID-mCherry-CENP-E cells were treated with 8 µM RO-3306 (Enzo Life Sciences) to enrich cells in G2 phase, followed by treatment with 5Ph-IAA for 2 h prior to imaging.

### Histologic observation

Whole-slide imaging of histological specimens was performed using a NanoZoomer S60v2 digital slide scanner (Hamamatsu Photonics) equipped with a 40 × objective lens. Images were acquired with NZAcquire software (Hamamatsu Photonics).

HE-stained archival cervical biopsy specimens were examined under IRB approval. All samples were anonymized, and informed consent was waived.

### Reverse transcription PCR

Total RNA was extracted using TRIzol reagent (Thermo Fisher Scientific) following DNase I treatment (Roche). First-strand cDNA was synthesized from the isolated RNA using the PrimeScript II 1st Strand cDNA Synthesis Kit (Takara Bio) with random primers. The resulting cDNA was used as a template for PCR amplification of E6 transcripts using primers described previously (30): forward, 5′-ATGCACCAAAAGAGAACTGC-3′; reverse, 5′-TTACAGCTGGGTTTCTCTACGTGT-3′.

### Structure prediction using AlphaFold3

Structural models of protein complexes were generated using AlphaFold3 via the DeepMind AlphaFold Server. For the trimeric p53-HPV16 E6-E6AP complex, residues 99–290 of human p53, 8–146 of HPV16 E6, and 126–761 of human E6AP were used as inputs. For modeling the CENP-E-E6-E6AP complex, the N-terminal 1-337 residues of human CENP-E were substituted for p53 in the same configuration. AlphaFold3 was run with default multimer settings, including full MSA and template search. Root-mean-square deviation (RMSD) between the predicted and experimentally determined structure of the p53-E6-E6AP complex was calculated using UCSF ChimeraX version 1.10.1.(37).

### Statistical analysis

Statistical analyses were performed using GraphPad Prism v10. Two-tailed Mann–Whitney tests were used to compare quantitative immunofluorescence intensities and mitotic durations. ECAC incidence was calculated for each independent experiment and compared using unpaired two-tailed Student’s *t*-tests when two groups were analyzed. For comparisons involving more than two groups, one-way ANOVA followed by Dunnett’s or Tukey’s multiple-comparisons test was used, as indicated in the figure legends. Chromosome-number variability was analyzed using the Kruskal-Wallis test followed by Dunn’s multiple-comparisons test. *P* values less than 0.05 were considered statistically significant. Box plots show the median and interquartile range, with individual data points overlaid.

## Data Availability

All data generated or analyzed during this study are included in this article.
